# Investigation of Electric Field–Induced Structural Changes at Fe-Doped SrTiO_3_ Anode Interfaces by Second Harmonic Generation

**DOI:** 10.3390/ma9110883

**Published:** 2016-10-31

**Authors:** David Ascienzo, Haochen Yuan, Steve Greenbaum, Thorsten J. M. Bayer, Russell A. Maier, Jian-Jun Wang, Clive A. Randall, Elizabeth C. Dickey, Haibin Zhao, Yuhang Ren

**Affiliations:** 1 Physics & Astronomy, Hunter College, the City University of New York, 695 Park Avenue, New York, NY 10065, USA; Dascienz@gmail.com (D.A.); yterminal@gmail.com (H.Y.); Steve.Greenbaum@hunter.cuny.edu (S.G.); 2 The Graduate Center, the City University of New York, 365 5th Ave., New York, NY 10016, USA; 3 Materials Science and Engineering, the Pennsylvania State University, University Park, PA 16802, USA; tjb5774@psu.edu (T.J.M.B.); russell.maier@nist.gov (R.A.M.); randall@matse.psu.edu (C.A.R.); 4 Materials Research Institute, the Pennsylvania State University, University Park, PA 16802; USA; jzw12@psu.edu; 5 Material Science and Engineering, North Carolina State University, Raleigh, NC 27695, USA; ecdickey@ncsu.edu; 6 Optical Science and Engineering, and Key Laboratory of Micro and Nano Photonic Structures (Ministry of Education), Fudan University, Shanghai 200433, China; hbzhao@fudan.edu.cn

**Keywords:** anodes, ceramics, electrostriction, oxides, perovskites, second harmonic generation

## Abstract

We report on the detection of electric field–induced second harmonic generation (EFISHG) from the anode interfaces of reduced and oxidized Fe-doped SrTiO_3_ (Fe:STO) single crystals. For the reduced crystal, we observe steady enhancements of the susceptibility components as the imposed *dc*-voltage increases. The enhancements are attributed to a field-stabilized electrostriction, leading to Fe:Ti-O bond stretching and bending in Fe:Ti-O_6_ octahedra. For the oxidized crystal, no obvious structural changes are observed below 16 kV/cm. Above 16 kV/cm, a sharp enhancement of the susceptibilities occurs due to local electrostrictive deformations in response to oxygen vacancy migrations away from the anode. Differences between the reduced and oxidized crystals are explained by their relative oxygen vacancy and free carrier concentrations which alter internal electric fields present at the Pt/Fe:STO interfaces. Our results show that the optical SHG technique is a powerful tool for detecting structural changes near perovskite-based oxide interfaces due to field-driven oxygen vacancy migration.

## 1. Introduction

Perovskite-type dielectric and ferroelectric oxides have attracted significant attention for their applications in a variety of electroceramic devices such as capacitors, thermistors, actuators, and sensors [1]. In order to improve the overall performance of these devices, it is important to understand breakdown and failure mechanisms brought on by *dc*-voltage and/or temperature stresses. Theoretical models based on defect chemistry and ionic transport point to the electromigration of oxygen vacancies as playing a dominant role in breakdown mechanisms such as aging, fatigue, and resistance degradation due to their relatively high concentration and mobility in perovskite oxides [2,3,4,5,6]. Under an applied *dc*-voltage, the oxygen vacancy concentration undergoes a demixing process in which oxygen ions migrate to the anode while oxygen vacancies migrate to the cathode. This demixing process occurs over shorter time scales at higher temperatures due to the lowering of the activation energy for oxygen vacancy diffusion [7]. In Fe-doped SrTiO_3_ (Fe:STO), Ti sites are partially filled by Fe atoms exhibiting mixed valence states of either Fe^3+^ or Fe^4+^ such that [Fe]_tot_ = [Fe^3+^] + [Fe^4+^] [8,9]. To conserve charge neutrality, oxygen vacancies form in the first coordination shell of Fe^3+^ centers. This leads to a larger oxygen vacancy concentration compared to undoped STO, making Fe:STO an excellent material for studying field-induced structural changes due to oxygen vacancy migration [5,10,11].

As a nonlinear spectroscopic technique, optical second harmonic generation (SHG) is a sensitive tool for probing local symmetry changes in dielectric and ferroelectric materials. Recent studies have used the optical SHG technique to probe polar domain microstructures in ferroelectric thin films [12,13,14], study the surface symmetry of intrinsic and Nb-doped SrTiO_3_ crystals [15], observe near-surface phase transitions in SrTiO_3_ [16], and detect internal electric fields due to oxygen vacancy ordering in ferroelectric films [17]. Most recently, optical SHG was used to study structural inhomogeneity at electrically degraded Fe:STO interfaces [18].

In this paper, we report on our detection of electric field–induced structural changes at reduced and oxidized Fe-doped SrTiO_3_ (Fe:STO) anode interfaces using optical SHG in the reflection geometry. For the reduced crystal, a gradual increase of the electric field-induced second harmonic generation (EFISHG) intensity is observed with an increasing *dc*-voltage. For the oxidized crystal, no obvious EFISHG intensity changes are identified below 16 kV/cm. When the *dc*-field further increases above 16 kV/cm, a sharp increase in the EFISHG intensity is revealed. The EFISHG intensity changes from both anode interfaces are explained by electrostrictive distortions to the crystal structure accompanied by oxygen vacancy migrations. Differences in the EFISHG responses are explained by the presence of internal electric fields at the Pt/Fe:STO interfaces which depend on local oxygen vacancies and free charge carrier concentrations. We show that optical SHG is a powerful tool for probing electric field–induced structural changes due to oxygen vacancy electromigration in perovskite oxides.

## 2. Experiment and Theory

The Verneuil-grown single crystals of SrTiO_3_ doped with 0.01 wt. % Fe were cut into 5 × 5 × 0.5 mm^3^ pieces and epi-polished on both sides ((100) orientation—MTI, Richmond, CA, USA). Then 10-nm-thick, amorphous platinum electrodes were sputtered on both planar sides for *dc*-field application. The details of the deposition conditions and the structural analysis, such as X-ray diffraction and cross-sectional transmission electron microscopy, were reported elsewhere [19]. To reduce or oxidize the single crystals, we annealed and equilibrated the crystals in a tube furnace at a temperature of 900 °C under an oxygen partial pressure (*p*O_2_) of 2 × 10^−5^ bar and *p*O_2_ of 0.2 bar, respectively. Then, reduced crystals were quenched in argon while oxidized crystals were quenched in air to freeze-in defect concentrations ([Fe] ~ 5.58 × 10^18^/cm^3^). As a result, reduced samples have a larger concentration of Fe^3+^ defect centers and oxygen vacancies (V_O_) while oxidized samples have a larger concentration of Fe^4+^ defect centers and fewer oxygen vacancies. The calculated defect concentrations of [V_O_], [Fe^3+^], and [Fe^4+^] based on mass action law constants are provided in Table 1 [9,20].

For optical SHG measurements, a mode-locked Ti:Sapphire ultrashort pulse laser (80 MHz, 10 nJ/pulse, 100 fs) was used as the fundamental light source (810 nm, ~10 µJ/cm^2^) with a focused beam spot diameter of ~10 µm on the sample. A Glan polarizer and band-pass filter (Thorlabs, FB400-40, Newton, NJ, USA) were placed in front of a photomultiplier tube module (Hamamatsu, H9305-04, San Jose, CA, USA) and connected to a lock-in amplifier (7265 DSP, Photonic Solutions, Edinburgh, UK) for signal detection.

**Figure 1:**
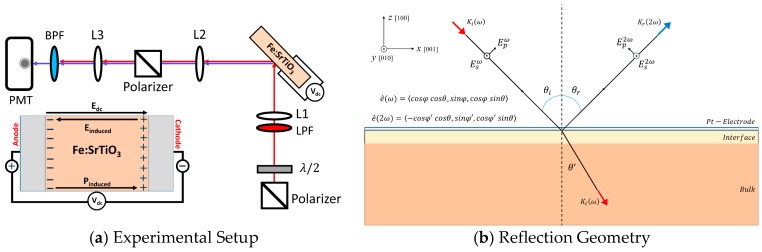
(**a**) Schematic for optical second harmonic generation (SHG) in the reflection geometry. Focusing lenses are marked by L1, L2, and L3. A band-pass filter (BPF) is used to block fundamental light from entering the photodetector (PMT). A long-pass filter (LPF) is placed before the sample to filter out residual SHG light from the laser system. A half-wave plate (λ/2) is used to rotate the polarization axis of the incident fundamental light. The insert shows the configuration with a *dc*-bias; (**b**) Schematic of the incident and outgoing light path and polarization for SHG measurements in the reflection geometry. (*x*, *y*, *z*) define the laboratory coordinates whereas [100], [010], and [001] represent crystal orientation. Incident and outgoing pulses travel along the *xz*-plane at an angle $\theta_{i}$ with respect to the surface normal and $\hat{e}\left(\omega \right)$ and $\hat{e}\left(2\omega \right)$ are the polarization directional unit vectors of the incident fundamental light and the outgoing SHG light. The *p*-polarized optical electric fields oscillate parallel to the *xz*-plane and *s*-polarized optical electric fields oscillate parallel to the *y*-axis.

As shown in Figure 1, the Fe:STO crystals were aligned with their normal axes at a tilt angle of θ= 45° with respect to the incident pump pulses traveling along the *xz*-plane. A half-wave plate was used to rotate the polarization angle, φ, of the incident pump pulses where φ is the angle between the polarization axis of the incident light and the *xz*-plane. Under an imposed *dc*-voltage, the reflected *p*-polarized (parallel to the *xz*-plane, φ=0°) and *s*-polarized (parallel to the *y*-axis, φ=90°) EFISHG intensity profiles were collected as functions of the incident polarization angle from the irradiated Pt/Fe:STO interfaces. In our study, a constant optical power is maintained so that all subsequent structural changes in the probed interfacial regions remain a function of the imposed *dc*-voltage.

At room temperature, the Fe:STO crystal bulk has predominately cubic (*Pm3m*) lattice symmetry. As a result of changes in the atomic packing order, inversion symmetry at the (100) surface is broken along the *z*-axis (normal axis) and the near-surface lattice transitions to tetragonal (*4mm*) symmetry [15,21]. The coherence length for SHG in the reflection (+) and transmission (−) geometries is expressed as [16]:

(1) $$L_{coh}=\frac{\lambda_{\omega }}{4\left|n^{\omega }\;\pm \;n^{2\omega }\right|}$$

where the coherence length represents the depth limit within the crystal that SHG radiation can be generated from. For the incident light of a wavelength of 810 nm, the coherence length in the reflection geometry is ~40 nm. Although the coherence length serves as an upper limit for the SHG probing depth, the actual probing depth in STO has been shown to be less than 1 nm [22]. As a result, the field-induced SHG response is dominated by electric-dipole and non-centrosymmetric defect contributions from the tetragonal (*4mm*) Pt/Fe:STO interface, while bulk contributions are suppressed [16]. Under an applied *dc*-voltage, within the dipole approximation, we can write the *i*-th Cartesian coordinate of the nonlinear polarization from the interface as follows:

(2) $$P_{i}\left(2\omega \right)=\epsilon_{o}\underset{jk}{\sum }\chi_{ijk}^{\left(2\right)}\left(2\omega ;\omega ,\omega \right):E_{j}^{loc}\left(\omega \right)E_{k}^{loc}\left(\omega \right)+\epsilon_{o}\underset{jkl}{\sum }\chi_{ijkl}^{\left(3\right)}\left(2\omega ;\omega ,\omega ,0\right)\vdots E_{j}^{loc}\left(\omega \right)E_{k}^{loc}\left(\omega \right)E_{l}^{loc}\left(0\right)$$

The first term accounts for interfacial dipole contributions to the SHG response in the absence of an external *dc*-field and the second term accounts for the *dc*-field–induced interfacial dipole contributions to the SHG response, i.e., EFISHG. Here $E^{loc}\left(\omega \right)=\overset{↔}{L}\left(\omega \right)\cdot E\left(\omega \right)$, where $\overset{↔}{L}\left(\omega \right)=L_{xx}^{\omega }\hat{i}\hat{i}+L_{yy}^{\omega }\hat{j}\hat{j}+L_{zz}^{\omega }\hat{k}\hat{k}$ is a diagonal tensor consisting of Fresnel transformation factors for light propagating at the fundamental frequency through the interfacial region and $E_{l}^{loc}\left(0\right)$ represents the *l*-th Cartesian coordinate of the *dc*-field inside the interfacial region. The non-vanishing components for the second-order and third-order susceptibility tensors in Equation (2) directly depend on the point group symmetry of the unit cells in the probed region. The third-order EFISHG term is reduced to a second-order term by taking *l* = *z* and integrating over the interfacial region such that we obtain:

(3) $$\underset{jk}{\sum }\left(\int \chi_{ijkz}^{\left(3\right)}\left(2\omega ;\omega ,\omega ,0\right)E_{z}^{loc}\left(0\right)dz\right):E_{j}^{loc}\left(\omega \right)E_{k}^{loc}\left(\omega \right)=\;\underset{jk}{\sum }\chi_{ijk}^{\left(2\right)}\left(2\omega ;\omega ,\omega \right)\left(E_{z}^{loc}\right):E_{j}^{loc}\left(\omega \right)E_{k}^{loc}\left(\omega \right)$$

The transformed second-order EFISHG susceptibility, $\chi_{ijk}^{\left(2\right)}\left(2\omega ;\omega ,\omega \right)\left(E_{z}^{loc}\right)$, has the same symmetry properties as $\chi_{ijk}^{\left(2\right)}\left(2\omega ;\omega ,\omega \right)$ for an imposed *dc*-field along the *z*-axis. Solving for P(2ω), the effective second-order susceptibility for the interface can be written out explicitly using [15,23,24]:

(4) $$\chi_{eff}^{\left(2\right)}=\langle \overset{↔}{L}\left(2\omega \right)\cdot \hat{e}\left(2\omega \right)\rangle \cdot \frac{P\left(2\omega \right)}{2\varepsilon_{o}{\left(E^{\omega }\right)}^{2}}$$

Here, the effective second-order susceptibility represents a function which depends on the tilt and rotational angles, θ and φ, of the incident optical fields (see Figure 1b), the imposed *dc*-voltage, and the non-vanishing susceptibility components of the interface. Further, $\overset{↔}{L}\left(2\omega \right)=L_{xx}^{2\omega }\hat{i}\hat{i}+L_{yy}^{2\omega }\hat{j}\hat{j}+L_{zz}^{2\omega }\hat{k}\hat{k}$ is a diagonal tensor consisting of Fresnel transformation factors for light propagating at the second harmonic frequency through the interfacial region and $\hat{e}\left(2\omega \right)$ is the polarization directional unit vector for the SHG electric fields reflected from the interface (Figure 1b).

The SHG intensities, $I_{p}^{2\omega }\left(\varphi \right)$ and $I_{s}^{2\omega }\left(\varphi \right)$, can then be written as functions of the incident light polarization angle, φ, by following $I^{2\omega }\propto {\left|E\left(2\omega \right)\right|}^{2}\propto \;{\left|\chi_{eff}^{\left(2\right)}\right|}^{2}$ and selecting outgoing SHG electric field polarizations either parallel to the *y*-axis (*s*-polarized) or the *xz*-plane (*p*-polarized) [24,25]:

(5) $$I_{p}^{2\omega }\left(\varphi \right)={\left|\chi_{pp}\;cos^{2}\varphi +\chi_{sp}\;sin^{2}\varphi \right|}^{2}$$

(6) $$I_{s}^{2\omega }\left(\varphi \right)={\left|\chi_{ds}\;sin\left(2\varphi \right)\right|}^{2}$$

where

$\chi_{sp}=\;\chi_{zxx}^{eff}L_{zz}^{2\omega }{\left(L_{yy}^{\omega }\right)}^{2}\sin\theta$
$\chi_{pp}=\;\chi_{zzz}^{eff}L_{zz}^{2\omega }{\left(L_{zz}^{\omega }\right)}^{2}sin^{3}\theta -2\chi_{xxz}^{eff}L_{xx}^{2\omega }L_{xx}^{\omega }L_{zz}^{\omega }\sin\theta cos^{2}\theta +\;\chi_{zxx}^{eff}L_{zz}^{2\omega }{\left(L_{xx}^{\omega }\right)}^{2}\sin\theta cos^{2}\theta$
$\chi_{ds}=\chi_{xxz}^{eff}L_{yy}^{2\omega }L_{yy}^{\omega }L_{zz}^{\omega }\sin\theta$

and

$\chi_{xxz}^{eff}=\;\chi_{xxz}+\chi_{xxz}\left(E_{z}^{loc}\right)$
$\chi_{zxx}^{eff}=\;\chi_{zxx}+\chi_{zxx}\left(E_{z}^{loc}\right)$
$\chi_{zzz}^{eff}=\chi_{zzz}+\chi_{zzz}\left(E_{z}^{loc}\right)$

## 3. Results and Discussion

**Figure 2:**
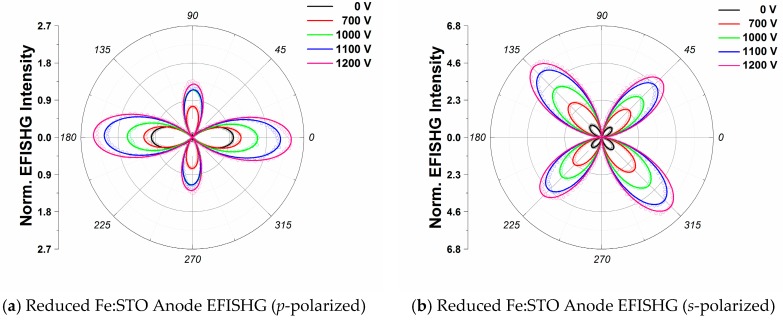
(**a**) The *p*-polarized EFISHG spectra for the reduced Pt-Fe:STO anode interface as a function of the incident pump polarization angle; (**b**) *s*-polarized EFISHG spectra for the reduced Pt-Fe:STO anode interface as a function of the incident pump polarization angle. *p*- and *s*- polarized curves are normalized to their respective 0 V signal peaks to show intensity gains. The solid lines show the theoretical fitting using Equations (5) and (6).

Figure 2a,b show the *p*-polarized and *s*-polarized EFISHG intensity profiles from the anode of the reduced Fe:STO sample. Solid lines show the fittings obtained using Equations (5) and (6) for *p*-polarized and *s*-polarized outgoing EFISHG intensities, respectively. We observe an apparent difference in magnitude between $I_{s}\left(45{}^{\circ}\right)$ and $I_{s}\left(135{}^{\circ}\right)$ at 0 V in the absence of an external *dc*-field which is fitted through the inclusion of a φ-independent term in Equation (6). These uneven peaks can be explained as an interference effect for *s*-polarized SHG light [26]. At 0 V it can be seen from Figure 2 that $I_{p}\left(0{}^{\circ}\right)$ is larger than $I_{s}\left(45{}^{\circ}\right)$ and $I_{s}\left(135{}^{\circ}\right)$; however, as the voltage is increased up to 1200 V, $I_{s}\left(135{}^{\circ}\right)$ becomes on par with, if not larger than, $I_{p}\left(0{}^{\circ}\right)$. Moreover, some small changes in the SHG intensity maxima positions are identified in both the *p*-polarized and *s*-polarized intensity profiles at higher voltages.

**Figure 3:**
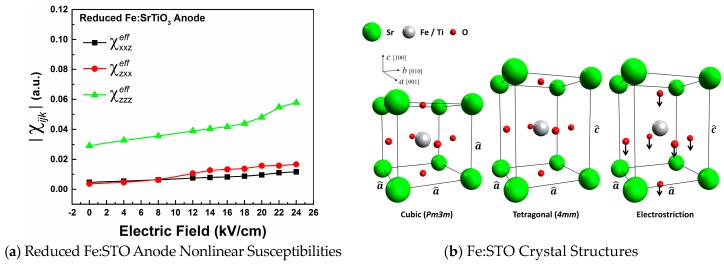
(**a**) The effective nonlinear susceptibility components as functions of the imposed *dc*-field for the reduced crystal’s anode interface; (**b**) Shows the cubic (*Pm3m*) unit cell, tetragonal (*4mm*) unit cell, and field-stabilized electrostrictive deformation of the tetragonal (*4mm*) unit cell for Fe:STO.

Figure 3a shows the effective susceptibility components plotted as functions of the imposed *dc*-voltage. Fittings show that $\chi_{zzz}^{eff}$ components are larger than both $\chi_{zxx}^{eff}$ and $\chi_{xxz}^{eff}$ for all voltages, as expected for an interface with tetragonal (*4mm*) symmetry. Previously, the field-induced structural changes to the nonlinear susceptibility components for the ABO_3_ crystal structure have been expressed as [27,28,29]:

(7) $$\delta \chi_{zzz}\left(E_{z}^{loc}\right)\propto \;{\left(d_{B-O}\right)}^{\sigma_{NL}}\left(\delta_{B}+\delta_{O\left(I\right)}\right)$$

(8) $$\delta \chi_{zxx}\left(E_{z}^{loc}\right)\propto \;\sigma_{NL}{\left(d_{B-O}\right)}^{\sigma_{NL}}\left(\delta_{B}+\delta_{O\left(II\right)}\right)$$

Here, $d_{B-O}$ is the Fe:Ti-O bond length, $\delta_{B}$ and $\delta_{O}$ are *dc*-field induced displacements for the B-site Fe:Ti cations and O-site oxygen anions, and $\sigma_{NL}$ is a factor related to the bond length, bond nonlinearity, and distortion of the O_6_ octahedron as discussed by Levine et al. [27,28]. In the polarizable bond-charge model, $\delta \chi_{zzz}\left(E_{z}^{loc}\right)$ correlates to a stretching of the *c*-axis Fe:Ti-O bonds along the *dc*-field axis (*z*-axis) while $\delta \chi_{zxx}\left(E_{z}^{loc}\right)$ correlates to a bending of the crystallographic *a*- and *b*-axis Fe:Ti-O bonds out of the *xy*-plane. These field-induced bond-bending and -stretching contributions to the SHG response can be interpreted as electrostrictive deformations, as shown in Figure 3b. Close to Kleinman’s symmetry considerations for a *4mm* structure, we would expect $\delta \chi_{zxx}\left(E_{z}^{loc}\right)$ and $\delta \chi_{xxz}\left(E_{z}^{loc}\right)$ to be approximately equal at lower voltages [29,30].

As oxygen vacancies migrate away from the anode interfacial region, tetragonally stretched Fe:Ti-O_6_ octahedra are left behind [9,31,32]. In response to the *dc*-field applied along the *z*-axis, O^2−^ anions shift towards the anode interface while Sr^2+^ and Ti^4+^/(Fe^3+^/Fe^4+^) cations shift away, resulting in significant ionic displacements as shown in Figure 3b. These ionic displacements involve a stretching (between the B-site cation and anode) and shortening (between the B-site cation and cathode) of Fe:Ti-O bonds along the *c*-axis, while transverse Fe:Ti-O bonds bend out of the crystallographic *ab*-plane. The ionic displacements lead to a dipole moment, or polarization, which is associated with the deformed unit cell. Tetragonal (*4mm*) symmetry of the Fe:Ti-O_6_ octahedron holds so long as the four Fe:Ti-O bonds along the *a*- and *b*-axis remain the same lengths, i.e., $\hat{a}=\hat{b}\neq \hat{c}$. It is important to note that electrostriction leads to an elongation of the crystal lattice along the applied field-axis. This elongation is identified from increases in $\chi_{zzz}^{eff}$ as the *dc*-voltage is increased.

**Figure 4:**
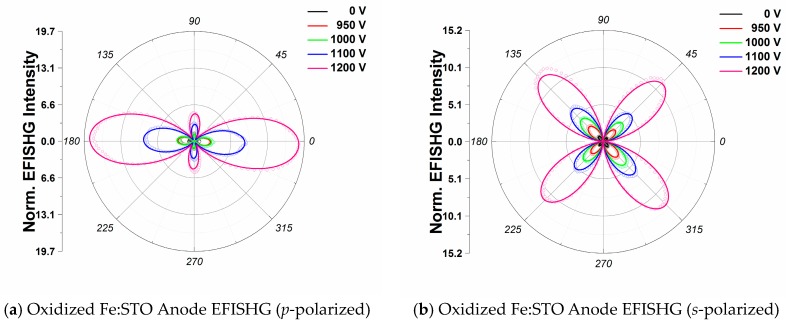
(**a**) *p*-polarized EFISHG spectra for the oxidized anode interface as a function of incident pump polarization angle; (**b**) *s*-polarized EFISHG spectra collected from the oxidized Pt-Fe:STO anode interface as a function of incident pump polarization angle. *p*- and *s*- polarized curves are normalized to their respective 0 V signal peaks to show intensity gains. The solid lines show the theoretical fitting using Equations (5) and (6).

For comparison, Figure 4a,b show the *p*-polarized and *s*-polarized EFISHG intensity profiles from the anode of the oxidized Fe:STO sample. Solid lines show the fittings obtained using Equations (5) and (6) for *p*-polarized and *s*-polarized EFISHG intensity profiles, respectively. Similar to the reduced crystal, we observe an apparent difference in magnitude between $I_{s}\left(45{}^{\circ}\right)$ and $I_{s}\left(135{}^{\circ}\right)$ which is fitted through the inclusion of a φ-independent term in Equation (6). Small changes in the EFISHG intensity maxima positions are also identified in both the *p*-polarized and *s*-polarized profiles at higher voltages.

**Figure 5:**
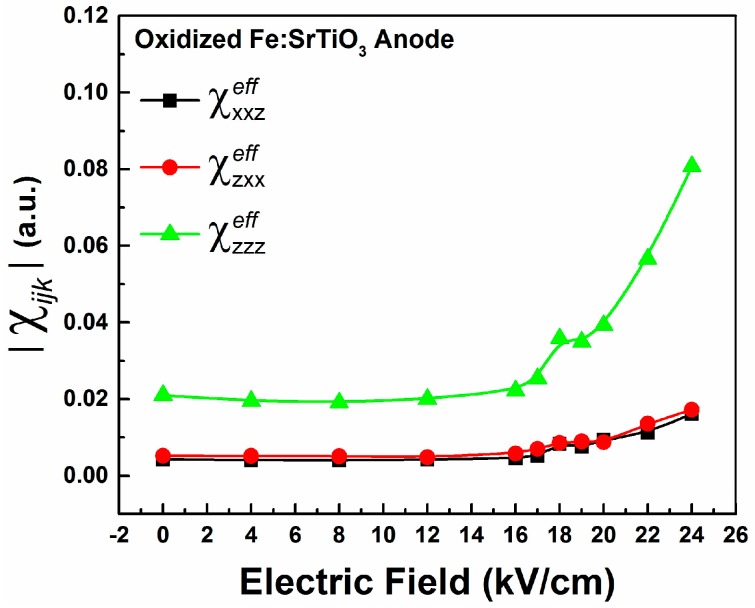
The effective nonlinear susceptibility components as functions of the imposed *dc* field for the oxidized crystal’s anode interface.

Figure 5 shows the effective susceptibility components as functions of the imposed *dc*-voltage for the oxidized Fe:STO anode interface. As expected, fittings also show that $\chi_{zzz}^{eff}$ components are larger than both $\chi_{zxx}^{eff}$ and $\chi_{xxz}^{eff}$ for all voltages; however, below 16 kV/cm, no obvious structural changes due to electrostriction are observed. Above 16 kV/cm, rapid enhancements of the EFISHG intensity, and therefore susceptibility components, are observed as shown in Figure 5. These changes are explained by a field-stabilized electrostriction effect. Around 16 kV/cm, a substantial migration of oxygen vacancies from the anode interface occurs and tetragonally stretched Fe:Ti-O_6_ octahedra are left behind. Differences between the EFISHG responses from the reduced and oxidized Fe:STO anode interfaces can be explained in accordance with the model substantiated by De Luca et al. [33] to explain EFISHG from LaAlO_3_/SrTiO_3_ interfaces. In their study, the EFISHG response is explained to arise from an internal electric field, generated by the accumulation of interfacial charged defects, which polarizes electronic orbitals [22,33]. For the case of Pt/Fe:STO, the Pt electrode behaves as an electron reservoir and positive charge (holes) accumulates between the Pt layer and the surface Fe:STO layer. As a result, oxygen anions migrate towards the anode while oxygen vacancies migrate to the cathode. Thus, the EFISHG response from the Fe:STO anode interface can be described in terms of an interfacial electric field which behaves as a barrier which opposes oxygen vacancy migration. The EFISHG responses show this interfacial field to be weaker at the reduced anode interface compared to the oxidized anode, resulting in stronger vacancy migration at lower *dc*-voltages. For the oxidized crystal, the interfacial field at the anode prevents oxygen vacancy migration until it is quenched by the external *dc* field near 16 kV/cm. These differences can then be attributed to the crystal’s defect concentrations since the reduced crystal has a higher number of oxygen vacancies and free charge carriers compared to the oxidized sample (see Table 1). In short, for higher vacancy concentrations, we observe greater electrostrictive distortions at lower *dc*-voltages. Our observations of structural changes are in good agreement with recent XRD studies performed by Hanzig et al. [32] and Li et al. [31] on SrTiO_3_ anodes. Both groups identified a lattice expansion along the *dc* field direction in response to oxygen vacancy migrations.

## 4. Conclusions

We employed the optical SHG technique to detect electric field–induced structural changes at the anode interfaces of reduced and oxidized Fe:STO single crystals. At the anode of the reduced Fe:STO sample, we observed steady enhancements of the susceptibility components as the imposed *dc*-voltage was increased. The enhancements are attributed to field-stabilized electrostriction, leading to Fe:Ti-O bond stretching and bending in Fe:Ti-O_6_ octahedra. At the anode of the oxidized Fe:STO sample, no obvious structural changes were observed below 16 kV/cm. Above 16 kV/cm, a sharp enhancement of the susceptibilities occurs and is explained by a quenching of the interfacial electric field by the external *dc*-field, allowing for local electrostrictive deformations to occur in response to oxygen vacancy migrations. Different EFISHG responses from the reduced and oxidized crystals are consistent with the higher oxygen vacancy concentration in the reduced sample. Our results show that the optical SHG technique is a powerful tool for detecting structural changes near perovskite-based oxide interfaces due to oxygen vacancy migration.

## Figures and Tables

**Table 1:** Defect concentrations.

pO_2_ (bar)	T (°C)	[V_O_] (cm^−3^)	[Fe^3+^] (cm^−3^)	[Fe^4+^] (cm^−3^)	[Fe^3+^]/[Fe]
0.2	900	1.03 × 10^18^	3.28 × 10^18^	2.30 × 10^18^	0.59
0.2	25	1.03 × 10^18^	2.06 × 10^18^	2.52 × 10^18^	0.37
2 × 10^−5^	900	2.43 × 10^18^	5.04 × 10^18^	5.44 × 10^17^	0.90
2 × 10^−5^	25	2.43 × 10^18^	4.85 × 10^18^	7.30 × 10^17^	0.87

Defect concentrations for the initial, undegraded states at the annealing (900 °C), quenching (25 °C), and investigation (25 °C) conditions.
